# Reformed Public Houses

**Published:** 1923-11

**Authors:** Fred Carter


### Reformed Public Houses.

SIR,—I am glad to see Mr. Dering's letter in the October issue. The importance of public house reform may well attract the attention of all who are concerned in promoting healthy conditions and who recognise that the existing public house fails entirely to cater in a healthy way for the public requirements.

Such a trade as this should properly be conducted in the public interest and ought not to be exploited for private gain. The People's Refreshment House Association should rightly receive appreciation for its pioneer work, and it is of interest to note that when the State management areas were instituted in 1916 many of its methods which had stood the test of experience were adopted.

Both in the City of Carlisle and in the rural area great attention is paid to the provision of substantial meals. Over half-a-million meals are served in the course of twelve months. The provision of counter-attractions is another feature, particularly in some of the villages.

The great merit of a public ownership scheme is that it deals on fair lines with the question of compensation to those who are disturbed by the transfer from private ownership. The absence of any such provision in most proposals for comprehensive reform renders them useless as practical remedies. Under the Carlisle scheme, the compensation to be paid was settled in 95 per cent. of the cases by negotiation, and only in 5 per cent. of the cases was it necessary to appoint an arbitrator.

FRED CARTER.

---

